# Adenovirus-36 Seropositivity and Its Relation with Obesity and Metabolic Profile in Children

**DOI:** 10.1155/2013/463194

**Published:** 2013-11-13

**Authors:** Isela Parra-Rojas, Oscar Del Moral-Hernández, Aralia B. Salgado-Bernabé, Iris P. Guzmán-Guzmán, Lorenzo Salgado-Goytia, José F. Muñoz-Valle

**Affiliations:** ^1^Laboratorio de Investigación en Obesidad y Diabetes, Unidad Académica de Ciencias Químico Biológicas, Universidad Autónoma de Guerrero, Avenida Lázaro Cárdenas S/N, Ciudad Universitaria, 39090 Chilpancingo, GR, Mexico; ^2^Laboratorio de Biomedicina Molecular, Unidad Académica de Ciencias Químico Biológicas, Universidad Autónoma de Guerrero, Avenida Lázaro Cárdenas S/N, Ciudad Universitaria, 39090 Chilpancingo, GR, Mexico; ^3^Departamento de Biología Molecular y Genómica, Centro Universitario de Ciencias de la Salud, Instituto de Investigación en Ciencias Biomédicas, Universidad de Guadalajara, Sierra Mojada 950, 44350 Guadalajara, JA, Mexico

## Abstract

The human adenovirus 36 (Ad-36) is causally and correlatively associated in animals and humans, respectively, with increased adiposity and altered metabolic profile. In previous studies, the relationship between Ad-36 seropositivity with obesity was established in adults and children. We evaluated the association of positive antibodies to Ad-36 with obesity and metabolic profile in Mexican children. Seventy-five children with normal-weight and 82 with obesity were studied in this research. All children had a clinic assessment which included weight, height, body circumferences, and skinfold thickness. Laboratory analyzes included triglycerides, total cholesterol, high-density lipoprotein, low-density lipoprotein, and glucose and insulin levels. An enzyme-linked immunosorbent assay (ELISA) was used to determine the antibodies to Ad-36 in the serum samples. The overall Ad-36 seroprevalence was 73.9%. Ad-36 seropositivity had a higher prevalence in obese children than in normal weight group (58.6 versus 41.4%, *P* = 0.007). Ad-36 seropositivity was associated with obesity (OR = 2.66, *P* = 0.01) and high-density lipoprotein <40 mg/dL (OR = 2.85, *P* = 0.03). The Ad-36 seropositive group had greater risk of 4 metabolic abnormalities compared with those children without none alteration. In summary, Ad-36 seropositivity was associated with obesity and low HDL-c levels in the sample of children studied.

## 1. Introduction 

Obesity has a complex, multifactorial etiology. Infectious agents have recently emerged as a possible contributor to the current obesity epidemic [1]. Considering the etiological role of infections in several other chronic diseases, a relationship between infections and obesity is plausible [2]. Adenovirus-36 (Ad-36) has been shown to cause obesity in chickens, mice, and nonhuman primates [3, 4]. It has been demonstrated that experimental and natural Ad-36 infection of multiple animal species resulted in obesity through increasing proliferation and differentiation of preadipocytes and lipid accumulation in mature adipocytes [3, 5, 6].

The data on association between Ad-36 and obesity in adults differ between studies being somewhat inconsistent, but the findings in children consistently associate Ad-36 infection with obesity. A study shown that 30% of obese and 11% of nonobese humans have neutralizing antibodies to Ad-36, and the presence of antibodies was associated with reductions in serum cholesterol and triglycerides [7]. In nondiabetic Swedish individuals, it was shown that Ad-36 infection is associated with pediatric obesity, severe obesity in adult females and lower risk of high blood lipid levels [8]. In a population of children in the United States, the prevalence of antibodies to Ad-36 was higher in obese children than in nonobese children. On average, antibody positivity was associated with 35-pound greater body weight [9]. In a group of obese school children from South Korea, 30% had antibodies to Ad-36, and infected children had higher body mass index *z*-scores than uninfected children [10]. Recent reports of adults in Italy and children in South Korea support the association of Ad-36 and obesity, and show that Ad-36 is more common in obese persons; prevalence ranges from 29% to 65% [11, 12].

In previous studies, the association of Ad-36 seropositivity with obesity was established in adults and children, but in Mexican population is unknown this relationship. The current study evaluated the association of positive antibody to Ad-36 with obesity and metabolic profile in a sample of Mexican children.

## 2. Materials and Methods

### 2.1. Participants

This research presents cross-sectional data from serum and clinical data that were collected between September and December, 2008. The sample included 75 normal-weight children and 82 obese children (*n* = 157, 6 to 11 years). The children were recruited of three schools in the urban area from Chilpancingo, state of Guerrero, Mexico. Informed written consent was obtained from all parents or guardians before the enrollment of children in the study. Approval for the study was obtained from the Research Ethics Committee of the University of Guerrero.

### 2.2. Clinic and Anthropometric Measurements

Body weight was determined in light clothes and without shoes using a Tanita body composition monitor (Tanita BC-553, Arlington, VA), and the height was measured to the nearest 0.1 cm using a stadiometer (Seca, Hamburg, Germany). From these measurements, body mass index (BMI) was calculated (BMI = weight/height^2^, kg/m^2^). The classification of normal weight and obesity was made using the 2000 Center for Disease Control and Prevention growth charts defining as normal weight, fifth to 85th percentiles and obesity, 95th percentile or higher. The body circumferences were measured in duplicate using a diameter tape accurate to within ±0.1 cm (Seca 201, Hamburg, Germany). The thickness of 4 skinfolds was measured to the nearest 0.1 mm, in duplicate, using skinfold caliper (Dynatronics Co, Salt Lake City, UT): triceps, biceps, subscapular, and suprailiac. The duplicate measures were averaged.

Blood pressure was measured on the right arm of children seated at rest for at least 5 minutes. Two consecutive measures were obtained at 1-minute intervals with an aneroid sphygmomanometer (Riester CE 0124, Jungingen, Germany). 

### 2.3. Laboratory Measurements

After overnight fasting, venous blood samples were collected. Biochemical parameters, such as LDL-cholesterol (LDL-c), total cholesterol, HDL-cholesterol (HDL-c), triglycerides (TG), and fasting glucose levels, were analyzed immediately using a semiautomated equipment (COBAS MIRA). Insulin levels were measured using a commercially available enzyme-linked immunosorbent assay (GenWay INS-EASIA kit). The HOMA index to determine insulin resistance was calculated using the formula [fasting insulin (*μ*U/mL) × fasting glucose (mmol/L)]/22.5. A qualitative determination using enzyme-linked immunosorbent assay (ELISA) was used to determine by duplicate the antibodies to Ad-36 in the serum samples (AdV36-Ab kit, Cusabio). We employed cut-off points from International Diabetes Federation proposal for children aged 10–16 years old for blood glucose [13], and cut-off points for plasma lipid and lipoprotein levels are from the NCEP Expert Panel on Cholesterol Levels in Children [14].

### 2.4. Statistical Analysis

Data analysis was performed using STATA software (V.9.2). Differences in characteristics between groups were evaluated using the Student's *t*-test for parametric variables and the Mann-Whitney *U* test for nonparametric variables. Multiple regression models were used to analyze the association of Ad-36 seropositivity with metabolic abnormalities. *P* < 0.05 was considered statistically significant.

## 3. Results

**Figure 1:**
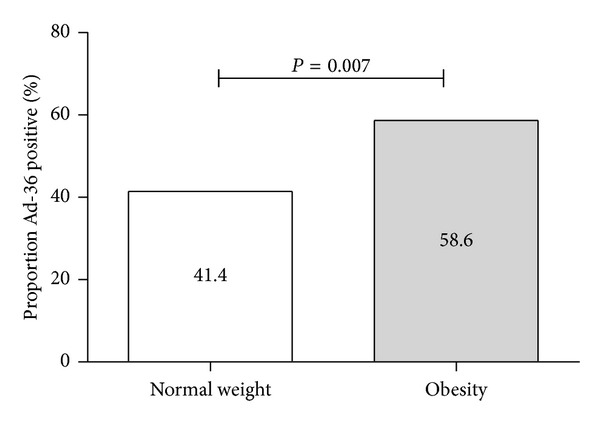
Ad-36 seropositivity in normal weight and obese children.

The overall Ad-36 seroprevalence was 73.9%. Among children with or without obesity, differences were present according to Ad-36 seropositivity (Figure 1). Ad-36 seropositivity had a higher prevalence in obese children than in normal weight group (58.6 versus 41.4%, *P* = 0.007).

A comparison between Ad-36 seropositive versus Ad-36 seronegative children is shown in Table 1. No significant differences in anthropometric and biochemical parameters were observed between groups. However, Ad-36 seropositive group resulted in a trend toward higher BMI (*P* = 0.08), systolic BP (*P* = 0.07), and total cholesterol (*P* = 0.09) and triglycerides levels (*P* = 0.09) than in Ad-36 negative group.

The effect of Ad-36 seropositivity on anthropometric and biochemical measurements was evaluated using the linear regression analysis, determining that the increase only of BMI (*β* = 1.58, *P* = 0.03), and triglycerides (*β* = 18.7, *P* = 0.05) may be explained by Ad-36 positivity (Table 2).

After adjustment for age and gender, Ad-36 seropositivity was associated with obesity (OR = 2.66, *P* = 0.01) and high-density lipoprotein <40 mg/dL (OR = 2.85, *P* = 0.03); other biochemical parameters were not related (Table 3).

Ad-36 seropositivity was analyzed with different number of metabolic alterations. The Ad-36 seropositive group had greater risk of 4 metabolic abnormalities compared with those children without none alteration (Table 4).

## 4. Discussion

In this study in Mexican children, significant association between Ad-36 seropositivity and obesity was found. The overall Ad-36 seroprevalence was 73.9% (41.4% and 58.6% in normal weight and obese children, resp.). Interestingly, a meta-analysis of 10 observational studies from around the world demonstrated that Ad-36 infection was associated with the risk of obesity and weight gain, but was not associated with abnormal metabolic markers including waist circumference [15].

Several studies determining the prevalence of Ad-36 antibodies in obese people have been carried out in North America, Sweden, Italy, Korea, and the Netherlands. In brief, a cross-sectional study in 124 children reported that Ad-36 positivity was present in 19 children (15%). Ad-36 positivity was significantly more frequent in obese children (15 [22%] of 67 children) than nonobese children (4 [7%] of 57 children) [9]. Another study found that 30% of obese and 11% of nonobese subjects have neutralizing antibodies to Ad-36 [7]. In Swedish individuals was show that Ad-36 infection is associated with pediatric obesity, severe obesity in adult females and lower risk of high blood lipid levels [8]. Also, Ad-36 seropositivity was assessed in 68 obese and 135 nonobese Italian subjects found that age, BMI, waist-hip ratio, blood pressure, insulin, HOMA, and triglycerides were significantly greater in the Ad-36 seropositive group [11]. In Korean schoolchildren, obese group have a higher prevalence of serum neutralizing antibodies to Ad-36 than nonobese group (28.57 versus 13.56%, resp.; *P* = 0.0174) [12]. In a study of 509 individuals from the Netherlands and Belgium, no significant association between Ad-36 seropositivity and obesity was found. The overall Ad-36 seroprevalence was 5.5% (3.9% and 5.7% in nonobese and obese subjects, resp.) [16].

In this study, the overall Ad-36 seroprevalence was higher (73.9%) compared with previous studies that have been carried out in USA, Korea, and Europe. Furthermore, it is very important to highlight that the prevalence found of Ad-36 positive was very high in children with obesity (58.6%), in comparison with previous reports. Consequently, our study supports the view that the Ad36-obesity association found in serum samples reflects obesity as a result of the infection, rather than the possibility that the Ad36-obesity association reflects an obese state being more susceptible to Ad-36 infection [8].

Several mechanisms have been postulated to explain the association between Ad-36 infection and obesity. Results of both *in vivo* and *in vitro* investigations have revealed that Ad-36 infection accelerates the differentiation of preadipocytes into adipocytes and their proliferation in studies of 3T3-L1 cells and human preadipocytes [3, 6, 17, 18]. Ad-36 infection also raises the lipid content of fat cells by promoting the uptake of lipids and glucose, which increases cellular lipid levels by stimulation *de novo* lipogenesis [19, 20]. Moreover, multiple factors ranging from genetics to biology and behavior may contribute to obesity in an individual and they may vary between individuals, making it difficult to isolate the relative contribution of any single factor. Therefore, the question of whether Ad-36 contributes to human obesity has remained incompletely answered, although some investigators seem to be progressing in the right direction [21].

The relationship of Ad-36 infection with abnormal metabolic parameters has also been analyzed. A meta-analysis of 10 observational studies demonstrated that Ad-36 infection was not associated with abnormal metabolic markers including total cholesterol, HDL-cholesterol, triglycerides, and glucose levels, systolic blood pressure, and waist circumference. Only a significant difference was found in relation to LDL-c [15].

Interestingly, we found an association of Ad-36 seropositivity with low HDL-c levels (OR = 2.85, *P* = 0.03), but other biochemical parameters were not related. Another study in the Korean schoolchildren, reported that within the obese group, the adjusted OR for the elevated triglycerides was significantly higher in Ad-36 antibody-positive children than those who were Ad-36 antibody-negative (OR = 2.328, 95% CI: 1.296–4.181) [12]. Similarly, Ad-36 was associated with lower serum lipids, cholesterol, and triglycerides (*P* < 0.003), in humans [7]. The explanation for this association is unknown. Some evidence from animal studies showed in golden Syrian hamsters that the cholesterol-lowering effect of Ad-36 infection is associated with a shift in plasma cholesterol from high-density lipoprotein to low-density lipoprotein cholesterol [22]. Another study, in wild-type (WT) mice and monocyte chemoattractant protein-1 knockout mice that were infected with Ad-36, found that Ad-36 infected WT mice increased insulin sensitivity, and lipid parameters such as HDL-c, LDL-c, and total cholesterol concentrations were lower than in mock-infected WT mice. The investigators assumed that MCP-1 regulates lipid metabolism in Ad36-induced obesity through inflammation by increasing the level of MCP-1 by means of the activation of nuclear factor *κ*B [23].

In our study, seropositive children to Ad-36 had not difference in glucose and insulin levels compared to seronegative children. In contrast, natural Ad-36 infection was cross-sectional associated with greater adiposity and better glycemic control in humans; in this study compared longitudinal observations of glycemic control (fasting glucose and insulin) in Ad-36 infected versus uninfected adults [24]. In muscle cells has been demonstrated that Ad-36 increased gene expression and protein abundance of GLUT1 and GLUT4, GLUT4 translocation to plasma membrane, and phosphatidylinositol 3-kinase (PI3-kinase) activity in an insulin-independent manner [25]; therefore may improve uptake of glucose and glycemic control in seropositive subjects.

The relationship between Ad-36 and nonalcoholic fatty liver disease (NAFLD) is being investigated. Ad-36 seropositivity was associated with a lower occurrence of NAFLD and bright liver, which, conceivably, is not directly mediated by insulin resistance [26]. Moreover, the role of Ad-36 in weight loss in NAFLD subjects with nutritional interventional treatment was studied. The subjects with previous infection have enhanced weight loss, bright liver disappearance, and recovery of insulin sensitivity [27]. Also, in another study it was associated with Ad-36 antibody status with response to a pediatric weight loss program in residential camp. Ad-36 antibody status showed a weak association with treatment response, but was associated with a better lipid profile. The authors suggest that Ad-36 antibody status should be assessed in studies of pediatric obesity treatment and prevention [28].

In Mexico, for the population in school age, from 5 to 11 years, the national prevalence combined of overweight and obesity in 2012 was 34.4% (19.8 and 14.6%, resp.) [29]. In our previous study, the prevalence of obesity and overweight in children were detected in 26.5% and 15.8%, respectively [30]; where the prevalence of obesity was 12% greater than the national prevalence. Therefore, one of the factors associated with the increase of obesity in children from Guerrero may be adenovirus 36 positivity. In humans, adenoviruses are frequently associated with acute upper respiratory tract infections, and they may also cause enteritis and conjunctivitis [2]. In Mexico, there is a lack of published data about the etiological agents causing acute respiratory infections; recently, it was reported that in a total of 100, children's nasopharyngeal samples showed a rate of adenovirus infection of 23%, and only Ad-C was detected [31]. In our study, children who were recruited had no symptoms of conjunctivitis or respiratory infection and nasopharyngeal samples were not obtained. Also, not asked whether children had these previous infections, therefore Ad-36 specific antibodies may remain detectable long after the initial infection. Moreover, Ad-36 seropositivity may indicate a persistent infection within obese children. Data for other adenovirus serotypes showed that antibodies reach undetectable levels within a 2-year period, but there are no available data on whether the time to reach undetectable levels is influenced by body weight [9].

## 5. Conclusion

In summary, this study provides evidence of the relationship of Ad-36 seropositivity with obesity and low HDL-c levels. Moreover, Ad-36 infection may contribute to increase the number of metabolic alterations in Mexican children.

## Figures and Tables

**Table 1:** Clinical and biochemical characteristics according to Ad-36 seropositivity.

Characteristics	Total *n* = 157	Ad-36 negative *n* = 41	Ad-36 positive *n* = 116	*P* value
Age (yr)	9 (6–11)	9 (6–11)	9 (6–11)	0.68
Gender				0.19
Male	75 (47.8)	16 (39.0)	59 (50.8)	
Female	82 (52.2)	25 (61.0)	57 (49.1)	
Weight (kg)	31.5 (20.4–59.1)	29.2 (20.4–58.5)	34.1 (20.7–59.1)	0.28
Height (cm)	132 (116.6–156)	132 (118–150)	131.5 (116.6–158)	0.38
BMI (kg/m^2^)	18.9 (14.4–26.6)	17.4 (14.8–25.19)	20.1 (14.4–26.6)	0.08
Systolic BP (mmHg)	98 (81–111)	95 (82–107)	99 (80–112)	0.07
Diastolic BP (mmHg)	58 (49–68)	58 (50–65)	59 (48–68)	0.40
Waist circumference (cm)	67 (55–88)	66 (56–86)	68.25 (55–88)	0.42
Arm circumference (cm)	21 (16.5–28)	20 (17–27)	21 (16–28)	0.40
Biceps skinfold (mm)	15.37 ± 4.64	15.52 ± 5.42	15.32 ± 4.36	0.83
Triceps skinfold (mm)	14.71 ± 4.03	14.87 ± 4.27	14.65 ± 3.96	0.77
Subscapular skinfold (mm)	14 (6–22)	13 (5.5–21.5)	14.75 (6–22)	0.44
Suprailiac skinfold (mm)	17 (9.5–26.5)	18 (8–25)	17 (9.5–29)	0.73
Total cholesterol (mg/dL)	179.1 ± 30.53	172.7 ± 27.45	181.5 ± 31.3	0.09
Triglycerides (mg/dL)	90 (36–200)	87 (36–161)	93 (36–200)	0.09
HDL-c (mg/dL)	53 (27–101)	55 (28–90)	52.5 (26–101)	0.26
LDL-c (mg/dL)	98.8 (59–174.6)	97.6 (52–159.8)	99.3 (60.9–179.5)	0.59
Glucose (mg/dL)	95 (75–112)	94 (73–109)	97 (79–112)	0.13
Insulin (*µ*U/mL)	6.6 (0.79–22.65)	6.37 (0.09–17.06)	6.72 (0.79–27.53)	0.49
HOMA	1.20 (0–5.47)	1.01 (0–4.08)	1.27 (0–6.64)	0.24

The parametric variables are shown means ± SD, median, and percentile 5 and 95 are shown for nonparametric variables. *P* values were obtained with the Student's *t*-test and the Mann-Whitney *U* test. *P* < 0.05 was statistically significant. BMI: Body mass index; HDL-c: high density lipoprotein-cholesterol; LDL-c, low density lipoprotein-cholesterol; HOMA: The Homeostasis Model Assessment.

**Table 2:** Effect of Ad-36 seropositivity on clinical and biochemical measurements.

Measurements	Without adjusted		Multiple models*	
	*β* (95% CI)	*P* value	*β* (95% CI)	*P* value
Weight (kg)	2.54 (−1.91–7.0)	0.26	3.18 (−0.45–6.8)	0.08
BMI (kg/m^2^)	1.46 (−0.02–2.95)	0.05	1.58 (0.12–3.04)	0.03
Systolic BP (mmHg)	2.67 (−0.66–6.0)	0.11	2.65 (−0.61–5.92)	0.11
Diastolic BP (mmHg)	0.50 (−1.82–2.82)	0.67	0.54 (−1.69–2.79)	0.63
Waist circumference (cm)	1.55 (−2.38–5.5)	0.43	1.83 (−1.82–5.48)	0.32
Arm circumference (cm)	0.54 (−0.81–1.89)	0.43	0.67 (−0.57–1.93)	0.28
Total cholesterol (mg/dL)	8.78 (−2.12–19.7)	0.11	8.71 (−2.32–19.7)	0.12
Triglycerides (mg/dL)	18.6 (0.05–37.1)	0.05	18.7 (−0.1–37.5)	0.05
HDL-c (mg/dL)	−3.24 (−11.5–5.0)	0.43	−2.97 (−11.3–5.34)	0.48
LDL-c (mg/dL)	6.0 (−7.2–19.2)	0.36	5.52 (−7.63–18.7)	0.40
Glucose (mg/dL)	2.95 (−0.76–6.67)	0.11	2.75 (−0.93–6.45)	0.14
Insulin (*µ*U/mL)	1.93 (−1.4–5.27)	0.25	2.09 (−1.28–5.47)	0.22
HOMA	0.52 (−0.17–1.22)	0.13	0.54 (−0.15–1.24)	0.12

Regression coefficient (95% CI). *Adjusted by age and gender.

**Table 3:** Association of Ad-36 seropositivity with metabolic abnormalities.

Characteristics	Ad-36 negative *n* = 41 (%)	Ad-36 positive *n* = 116 (%)	*P* value	*OR (IC 95%) *P* value
BMI			0.007	
Normal Weight	27 (36.0)	48 (64.0)		1.0
Obesity	14 (17.1)	68 (73.9)		2.66 (1.26–5.63) *P* = 0.01
Glucose			0.19	
<100 mg/dL	31 (75.6)	75 (64.6)		1.0
≥100 mg/dL	10 (24.4)	41 (35.4)		1.68 (0.74–3.82) *P* = 0.21
Cholesterol			0.28	
<170 mg/dL	18 (43.9)	40 (34.5)		1.0
≥170 mg/dL	23 (56.1)	76 (65.5)		1.49 (0.71–3.1) *P* = 0.28
Triglycerides			0.21	
<90 mg/dL	23 (56.1)	52 (44.8)		1.0
≥90 mg/dL	18 (43.9)	64 (55.2)		1.53 (0.74–3.1) *P* = 0.24
HDL-c			0.026	
≥40 mg/dL	35 (85.4)	78 (67.2)		1.0
<40 mg/dL	6 (14.6)	38 (32.8)		2.85 (1.09–7.4) *P* = 0.03

*Logistic regression adjusted by age and gender.

**Table 4:** Ad-36 seropositivity and number of metabolic abnormalities.

Characteristics	Ad-36 negative *n* = 41 (%)	Ad-36 positive *n* = 116 (%)	*OR (IC 95%) *P* value
None	10 (24.4)	15 (12.9)	1.0
1	10 (24.4)	22 (19.0)	1.52 (0.50–4.62) *P* = 0.45
2	9 (21.9)	20 (17.2)	1.34 (0.42–4.21) *P* = 0.61
3	7 (17.1)	25 (21.5)	2.56 (0.78–8.4) *P* = 0.12
≥4	5 (12.2)	34 (29.3)	4.36 (1.25–15.1) *P* = 0.02

*Logistic regression adjusted by age and gender.
